# Experiences of Health Professionals Regarding Existing Guidelines Used to Manage Obstetric Emergencies in a Rural Area of South Africa: A Qualitative Explorative Study

**DOI:** 10.3390/ijerph23050555

**Published:** 2026-04-25

**Authors:** Caroline Sindisa Baloyi, Cairo Bruce Ntimana, Eric Maimela

**Affiliations:** 1 Department of Public Health, University of Limpopo, Sovenga St., Polokwane 0727, South Africa; sindisa545@gmail.com; 2 DIMAMO Population Health Research Centre, University of Limpopo, Sovenga St., Polokwane 0727, South Africa; 3 Department of Public Health, Walter Sisulu University, Mthatha 5117, South Africa; emaimela@wsu.ac.za

**Keywords:** maternal mortality, obstetric emergencies, health systems strengthening

## Abstract

**Highlights:**

**Public health relevance—how does this work relate to a public health issue?**

- The study identifies critical gaps between policy and practice that directly contribute to high maternal and neonatal mortality rates.
- The research provides evidence to help policymakers tailor guidelines to the rural context rather than applying a one-size-fits-all approach, ensuring that guidelines are relevant to the local environment.

**Public health significance—why is this work of significance to public health?**

- The study directly addresses a critical gap in reducing maternal and neonatal mortality in underserved regions.
- The study provides ideas on tailoring guidelines to rural realities and helps in developing or adapting guidelines that are practical and context-specific, rather than forcing urban-centric protocols onto rural settings.

**Public health implications—What are the key implications or messages for practitioners, policy makers and/or researchers in public health?**

- Reducing high maternal and neonatal mortality by addressing gaps in the “three delays” model.
- Providing evidence to tailor national guidelines to the practical realities of rural, low-resource settings, thereby improving the quality of care and reducing maternal and perinatal mortality.

**Abstract:**

Despite the availability of clinical guidelines aimed at managing pregnancy complications, maternal deaths related to obstetric emergencies remain unacceptably high in South Africa, especially in rural provinces like Limpopo. These preventable deaths are often linked to delayed response to complications, poor adherence to protocols, and lack of essential resources. The study aimed to explore the experiences of health professionals regarding the implementation of maternal guidelines used to manage obstetric emergencies. The study adopted a qualitative, descriptive, and explorative design. Data were analysed thematically, and trustworthiness was maintained throughout the research process. Sixteen participants from four selected hospitals in a rural area of South Africa (Vhembe District, Limpopo Province) were purposively sampled and interviewed using semi-structured interviews; data were analysed thematically. The findings highlighted multiple critical barriers to guideline implementation, including shortages of printed clinical protocols, inconsistent patient follow-up, poor referral systems, infrastructure deficits, medication stock-outs, and negative staff attitudes. Most doctors and midwives working in maternity units lacked training on the Essential Steps in the Management of Obstetric Emergencies (ESMOE), resulting in insufficient knowledge and skills to manage obstetric emergencies. Therefore, there is an urgent need for comprehensive ESMOE training for all doctors and midwives in maternity units.

## 1. Introduction

Globally, leading causes of maternal mortality include post-partum haemorrhage, hypertensive disorders such as eclampsia, sepsis, obstructed labour, and uterine rupture [1,2]. While improvements have been noted in the management of obstetric haemorrhage and non-pregnancy-related infections, preventable deaths continue to occur due to delays in care, health system weaknesses, and substandard implementation of clinical guidelines [3,4,5]. Although evidence-based maternal health guidelines are widely available and intended to support healthcare workers in managing obstetric emergencies, their effective implementation and monitoring remain inconsistent, particularly in resource-limited and rural settings [6,7].

In South Africa, maternal mortality has declined over recent years; however, the country has not yet achieved Sustainable Development Goal 3 (SDG 3), which aims to ensure good health and well-being for all by 2030, including reducing the maternal mortality ratio to fewer than 70 per 100,000 live births [8]. The institutional maternal ratio decreased from 148 to below 100 per 100,000 live births [9,10]. Despite this process, significant disparities persist. Limpopo Province reported a maternal mortality ratio (MMR) of 130 per 100,000 live births, contributing 10.5% of the national maternal mortality burden [11]. Given that Limpopo represents approximately 10% of South Africa’s population, its 10.5% contribution to national maternal deaths is proportionate rather than disproportionate. However, when compared to the national institutional MMR (below 100 per 100,000 live births), Limpopo’s MMR of 130 remains substantially higher, indicating a persistent rural disadvantage [10,11]. This pattern is not unique to Limpopo; other predominantly rural provinces, such as the Eastern Cape and KwaZulu-Natal, have similarly reported MMRs exceeding the national average, reflecting broader challenges facing rural health systems across South Africa [11]. Internationally, rural and resource-limited settings in sub-Saharan Africa consistently demonstrate higher maternal mortality ratios compared to urban centres, with barriers including geographic inaccessibility, health worker shortages, and weak referral systems [1,2].

High maternal mortality rates have been observed among woman residing in rural and socioeconomically disadvantaged communities, where access to quality maternal healthcare services remains limited [12].

This study offers several novel contributions. First, while previous research has documented barriers to obstetric care in Limpopo, no study has focused specifically on the Vhembe district, a remote area bordering Zimbabwe and Mozambique with persistently high maternal mortality as reported by the district health information system [13]. Second, by including both doctors and midwives from the same facilities, we capture interprofessional dynamics that affect guideline adherence, an aspect under-explored in previous research that has focused primarily on midwives alone. Third, rather than merely listing barriers, we explicitly map each barrier to specific guideline requirements, revealing the guideline–practice gap. Fourth, our data were collected in 2024–2025, providing an updated post-pandemic assessment of health system challenges, including staff attrition and resource constraints that may have intensified since earlier studies were conducted**.**

Despite the availability of national maternal health guidelines in South Africa, preventable maternal deaths continue to occur, often linked to substandard care and challenges in adherence to guidelines. [14,15]. While systems to reduce maternal mortality exist, inadequate monitoring, limited resources, and contextual barriers hinder effective implementation [16].Therefore, the following research questions guided this study: (1) What are the experiences of healthcare professionals regarding the implementation of existing guidelines for managing obstetric emergencies in rural healthcare facilities? (2) What barriers do healthcare professionals perceive as hindering effective guideline implementation? (3) What recommendations do healthcare professionals offer to improve guideline implementation in rural settings?.

This study aimed to explore and describe the experiences and perceptions of healthcare professionals (doctors and midwives) regarding the implementation of existing clinical guidelines for managing obstetric emergencies in rural areas of the Vhembe district, Limpopo Province.

## 2. Materials and Methods

### 2.1. Study Design

A qualitative approach using descriptive and explorative methods was adopted. A qualitative approach fostered interaction between the researcher and the participants. During this interaction, the topic was relevant to this paper since the experience of healthcare professionals regarding existing guidelines to manage obstetric emergencies in rural areas in South Africa was explored. Each health professional was awarded an opportunity to describe their experiences without any limitations.

### 2.2. Setting

This study was conducted in the Vhembe district of Limpopo Province; the Vhembe district is situated in the northern part of Limpopo Province. It is primarily rural. There are eight health facilities in the Vhembe district. The researcher chose four facilities as they reported a high number of maternal mortalities from 2018 to 2023, as reported by the district health information system [13].

### 2.3. Recruitment and Sampling of Participants

After obtaining permission to conduct the study from the Chief Executive Officers of the four hospitals, the operational managers who were responsible for facilitating the meetings with the doctors and midwives were requested to assist with arranging the meetings. During the meetings, the participants were informed about the study’s purpose. It was clearly explained to the participants that not all of them would be included in the study, as a certain number of participants is required. The inclusion criteria were clearly explained to them. Purposive sampling was used to select participants. The following characteristics guided the sampling process: (1) profession—doctors and midwives working in maternity departments, as they were most relevant to the study objectives; (2) minimum of two years’ experience in the maternity ward, ensuring participants had sufficient exposure to obstetric emergencies; (3) willingness to share their experiences regarding the implementation of maternal guidelines. These criteria were chosen to ensure that participants possessed the necessary knowledge, experience, and insight to provide rich, relevant data on the barriers to and facilitators of guideline implementation in rural obstetric care settings. The researcher selected doctors and midwives working in those hospitals who happened to have the information to respond to the objectives of the study.

### 2.4. Inclusion and Exclusion

Participants were health professionals (doctors and midwives) working in a maternity ward with two years’ experience. These consisted of midwives with basic midwifery or advanced midwifery, and they were working in any of the four selected health facilities. Doctors and midwives who refused to participate in the study were excluded, as well as those with less than two years of experience.

### 2.5. Data Collection

Data were collected in all four selected health facilities. The researcher visited the participants at the hospitals before data collection to build trust as a way of ensuring prolonged engagement. Informed consent was obtained from the participants, and informed consent to use a tape recorder was obtained. The reasons to use a tape recorder were given; this will enable the researcher to capture everything said by the participants. Face-to- face interviews were conducted with doctors and midwives, who were purposively sampled by the researcher. The interview guide was piloted with two healthcare professionals (one doctor and one midwife) from a non-participating hospital in the same district. No modifications were needed, and these pilot interviews were not included in the final analysis.

All interviews were conducted by the first author (C.S.B.), a female public health researcher with experience in maternal health services. She did not work in any of the four participating hospitals and had no prior supervisory or employment relationship with the participants. Participants were informed that she was an independent researcher from the University of Limpopo, which helped to minimise social desirability bias. Interviews were conducted in a private room within each hospital (e.g., a vacant office or consultation room) to ensure privacy. No hospital staff other than the participant and researcher were present during interviews.

The study was guided by one central question: “**What are your experiences and perceptions regarding the implementation of existing guidelines for managing obstetric emergencies in this facility?**” This was followed by probing questions depending on the participant’s response. Data were collected until data saturation, whereby the participants were given the same information. The researcher has ensured that no physical, psychological, or emotional harm has been caused to the participants. The questions were asked in an appropriate manner, and participants were not judged. At the end of each interview, the tape recorder was played back for the participants to verify the recorded information and identify gaps. Data were collected in English and then transcribed verbatim. Each interview lasted 30–45 min.

### 2.6. Data Analysis

The Tesch approach by Creswell was used to analyse data gathered from doctors and midwives conducted during face-to-face interviews [17]. The analysis was performed by the researchers collectively in scheduled group meetings. Three researchers were involved in the analysis: the first author (C.S.B.), a female public health researcher with experience in maternal health services; the second author (C.B.N.), a researcher with expertise in qualitative and quantitative methodologies; and the third author (E.M.), a Professor of Epidemiology with extensive qualitative research experience. All three researchers held graduate degrees and had prior experience conducting thematic analysis. No qualitative data analysis software was used. Analysis was conducted manually using Microsoft Word for transcript management and Excel for coding and categorisation. The following eight-step process was followed. Step 1: Each researcher independently read all transcripts multiple times to gain an overall understanding of the content. Marginal notes were made to capture initial impressions. Step 2: One transcript was selected for detailed analysis. Each researcher identified underlying meanings and wrote these ideas in the margins. Step 3: After individually analysing the first transcript, the research team met to discuss the identified topics. Similar topics were grouped together, and a preliminary list of topics was created. Step 4: This list of topics was then applied to a second transcript. The researchers independently coded this transcript using the preliminary topics, adding new topics as they emerged. Step 5: The research team met again to compare coding results from the second transcript. Discrepancies were discussed and resolved by consensus. Topics were refined, merged where similar, and separated where distinct. Step 6: Using the refined coding framework, each researcher independently coded all remaining transcripts. Step 7: The team convened to review all coded data. Related topics were collapsed into subcategories, and subcategories were organised into broader categories. This process involved multiple rounds of discussion and reorganisation. Step 8: Finally, the categories were reviewed to identify overarching themes that represented the major findings of the study.

The supervisor served as an independent coder who cross-checked the themes and categories. The inter-coder agreement process involved the following: after each researcher completed independent coding, the supervisor compared the coding results. Areas of disagreement were discussed in team meetings until a consensus was reached. No formal inter-coder reliability statistic (e.g., Cohen’s kappa) was calculated, as this study followed a qualitative descriptive approach where consensus-building through discussion is considered appropriate. The authors then interpreted the findings of the study, explaining what they had learned regarding the management of obstetric emergencies.

### 2.7. Trustworthiness

Trustworthiness was used, which measured the quality of the research [18]. Four criteria were addressed: credibility, transferability, dependability, and confirmability.

**Credibility** was established through member checking. After each interview, the researcher played back the recorded audio to the participant to verify the information. Participants confirmed that their views had been accurately captured. The researcher’s interpretations and conclusions were also validated by the research team.

**Transferability** was ensured by providing a detailed description of the research process. This includes the study design, setting, population, sampling procedure, and data collection methods. These rich descriptions allow readers to assess whether the findings may apply to other similar contexts.

**Dependability** was achieved through consultation with the research team and supervisors. Regular meetings were held to review the data and analysis. This ensured consistency in how data were collected and recorded. The researcher maintained an accurate record of all steps taken during the study.

**Confirmability** was ensured by playing back recorded audio during interviews and transcribing the data verbatim. Participants verified the transcriptions to confirm that their responses were represented accurately. The researcher also kept reflective notes to minimise personal bias.

**Consensus process transparency:** To make sure that the results were based on participant data, not on the personal biases of the researchers involved in the study, the following consensus process was used. After each researcher individually codes the transcripts, three consensus meetings are held by the team. In the first meeting, every researcher presents the coding framework they have created, and discrepancies between them are noted. The second meeting is dedicated to solving any disagreements between the coders using discussion and comparing every problematic code back to the transcript. Consensus is achieved when all discrepancies are discussed and resolved. In the third meeting, subcategories and themes are defined. An independent coder, the supervisor, takes part in all the meetings and provides an objective view. The minutes of the meetings are taken.

**Reflexivity and positionality:** The research team acknowledged that their backgrounds and worldviews could influence data collection, analysis, and interpretation. The first author (C.S.B.) is a female public health researcher with experience in maternal health services in Limpopo Province. She has worked closely with midwives and doctors in rural facilities, which provided an insider understanding of the context but also required conscious effort to bracket preconceptions. To minimise bias, she maintained a reflective journal throughout the study, documenting personal assumptions and how they might shape interactions with participants. The second author (C.B.N.) is a male population health researcher at the DIMAMO Population Health Research Centre (PHRC) at the University of Limpopo. His research experience includes conducting qualitative and quantitative studies, data quality control, and biostatistics. His expertise in both qualitative and quantitative research methodologies, along with his experience in health systems research in rural Limpopo, provided valuable insight during data analysis and interpretation.

The third author (E.M.) is a male public health specialist who holds a PhD in Medical Sciences (UL), a Doctor of Medical Sciences (UA), an MSc in Epidemiology (UP), a Diploma in Health Services Management (UP), and a BSc in Medical Sciences (UL). He is a Professor in Epidemiology in the Department of Public Health, Faculty of Medicine and Health Sciences at Walter Sisulu University. His expertise in epidemiological methods and qualitative research provided critical oversight in study design, data analysis, and interpretation.

Both the second and third authors served as independent auditors, reviewing coding decisions and challenging interpretations to ensure that findings were grounded in participant data rather than researcher assumptions. Regular team debriefing sessions were held to critically examine how each researcher’s professional background and disciplinary lens might influence data analysis. This reflexive process enhanced the authenticity of the findings.

## 3. Results

The study included 16 participants, comprising doctors and nurses with varying years of professional experience. Eight participants were medical doctors (four males and four females) with experience ranging from 2 to 13 years. The remaining eight participants were nurses, all female, including three advanced midwives, with experience ranging from 3 to 17 years. Participants’ ages ranged from 32 to 55 years, reflecting a diverse and experienced healthcare workforce (Table 1).

Four main themes emerged, namely the experiences of healthcare professionals regarding existing guidelines to manage obstetric emergencies and the perceptions of health professionals on the management of obstetric emergencies; implementation of maternal care guidelines; lack of resources; and the competency of staff and health-seeking behaviour (Table 2).

**Theme 1: Experiences of health professionals regarding existing guidelines used to manage obstetric emergencies**

This theme captures participants’ direct reflections on the maternal clinical guidelines currently available in their facilities. The subthemes below illustrate how the practicality, scope, and content of the guidelines influence healthcare professionals’ ability to use them effectively during obstetric emergencies.

**Subtheme 1.1: Practicality of guidelines**

This subtheme was mentioned by 3 out of 16 participants (19%). The study revealed that it is not practical to implement some of the items in the guideline, wherein a doctor is expected to respond within 15 min of being called to attend to the patient. This finding directly relates to participants’ perceptions of the guidelines, as they identified specific guideline requirements that are unrealistic in their rural facility context. The following quotes depict how the implementation of guidelines is not practical:

Participant 1 (male doctor, 13 years of experience) said: “*A doctor shall respond within 15minutes of being called to attend the patient. This is not practical in some facilities, as there are no call rooms, doctors had to walk a distance to respond to emergency.*”

Participant 5 (male doctor, 11 years of experience) said: “*Maternity guideline is structured in a manner that it addresses theory not the practical part of it, during emergency it is difficult to open the guideline and start reading, but you need to act immediately to save life.*”

Participant 8 (male doctor, 2 years of experience) said: “*There is no consultation done before maternity guideline was released for implementation.*”

**Subtheme 1.2: Guideline-imposed limitations on nursing scope of practice**

This subtheme was mentioned by 2 out of 16 participants (13%). Participants reported that the existing maternal guidelines restrict nurses from initiating critical emergency treatments without a doctor’s order. While this reflects the formal scope of practice defined in the guidelines, participants perceived these restrictions as a barrier to timely emergency care. The following quotations illustrate how the guidelines themselves create delays in management:

Participant 16 (advanced midwife, 8 years of experience) said: “*I had a pregnant woman with gestational hypertension, I managed to stabilise the patient by doing the basics, such as monitoring fetal heart using doppler, putting a woman on CTG, monitoring vital sign, checking urine, but I cannot give treatment as I must wait for the doctor to prescribe treatment. This delays early intervention to manage the management.*”

Participant 14 (advanced midwife, 10 years of experience) said: “*I was having a patient with excessive bleeding and having complications, the patient was having placenta praevia, I could see the woman cannot deliver vaginally but needs to be done caesarean section immediately, but the doctor will say continue progressing labour, the woman bled profusely and after an hour she was then taken to theatre and she delivered a fresh still birth, then the woman was taken to ICU, as she had bleed more and she needed a critical care, but this could have been avoided if the scope of practice does allows advanced midwife to take a decision on the mode of delivery especially at the hospital level.*”

**Subtheme 1.3: Delayed seeking help (a contextual barrier to guideline implementation)**

This subtheme was mentioned by 1 out of 16 participants (6%). While not directly about the guidelines themselves, this subtheme represents a critical external factor that shapes whether guidelines can be followed. When patients arrive very late, healthcare professionals cannot implement guideline-recommended interventions because the clinical situation has already progressed beyond the point where standard protocols are effective. The findings of the study revealed that some patients arrive very late at the hospital, whereby the progression of illness cannot be managed because it is already late. These findings are supported by the following quotations:

Participant 2 (female doctor, 2 years of experience) said: “*One of the pregnant woman who was 18 weeks pregnant died of ectopic pregnancy, a woman had a history of severe abdominal pain, had vaginal bleeding, for a week and she never intended to seek help immediately, she arrived in casualty diagnosed ectopic pregnancy but unfortunate that the patient died while being resuscitated in casualty.*”

**Subtheme 1.4: Defaulted treatment (a contextual barrier to guideline implementation)**

This subtheme was mentioned by 1 out of 16 participants (6%). This subtheme reflects patient behaviours that occur before woman reach the facility. When pregnant woman default on treatment or fail to attend antenatal care, healthcare professionals inherit clinical situations that deviate from the assumptions embedded in standard guidelines (which assume patients are engaged in ongoing care). Participants perceived this as a barrier to following guideline-recommended pathways. The findings of the study revealed that pregnant woman who default on treatment face serious health risks that lead to maternal deaths. These findings are supported by the following quotations:

Participant 6 (female doctor, 4 years of experience) said: “*A pregnant woman at 38 weeks, was diagnosed with hypertension before she falls pregnant, she was started with hypertensive drugs, when she felt that she is pregnant, she never initiated ANC visit, she did not book an ANC visit, she defaulted on treatment for hypertension, she started having eclampsia, she got admitted in the hospital, a caesarean section was done, she had an intra-uterine fetal death, immediately after delivering the baby she had post-partum haemorrhage and died while in recovery room.*”

**Subtheme 1.5: Attitude of staff (an interpersonal barrier to guideline adherence)**

This subtheme was mentioned by 1 out of 16 participants (6%). This subtheme captures how interpersonal dynamics between healthcare professionals affect adherence to guidelines. When midwives undermine junior doctors or refuse to follow their instructions, the result is deviation from guideline-recommended management. Thus, staff attitudes function as a barrier to consistent guideline implementation. The findings of the study revealed that specialists undermine doctors who are transferring patients to regional and tertiary hospitals. These findings are supported by the following quotations:

Participant 6 (female doctor, 4 years of experience) said: “*Some of the midwives are very arrogant and feel that the doctors do not know how to manage obstetric emergencies. They undermine doctors especially intern doctors, they refuse to carry instructions from the doctors saying the management is wrong.*”

**Theme 2: Implementation of maternal care guidelines**

This theme focuses directly on the processes and systems that enable or prevent healthcare professionals from following established protocols. The subthemes below describe training deficits and staffing shortages as key barriers to putting guidelines into practice.

**Subtheme 2.1: Lack of essential steps in managing obstetric emergencies (ESMOE) training**

This subtheme was mentioned by 2 out of 16 participants (13%). This subtheme is central to the study aim, as ESMOE is the primary training framework designed to operationalise maternal guidelines in South Africa. Without ESMOE training, participants cannot translate guideline recommendations into clinical action. The findings of the study revealed that health professionals are not taught anything on ESMOE guidelines either at the clinics and hospitals due to a lack of resources to facilitate training. These findings are supported by the following quotations:

Participant 2 (female doctor, 2 years of experience) said: “*All obstetric emergencies must be treated following ESMOE and Limpopo obstetrics and gynaecology protocols and standards. Only few health professionals are trained ESMOE, which is not practical to implement the protocols due to lack of ESMOE training.*”

Participant 6 (female doctor, 4 years of experience) said that: “*All obstetric emergencies cases must be treated following ESMOE and Limpopo obstetrics and gynaecology protocols and standards, but only few health professionals are trained ESMOE.*”

**Subtheme 2.2: Shortage of staff (a health system barrier to guideline implementation)**

This subtheme was mentioned by 2 out of 16 participants (13%). While staff shortages are not directly about the guidelines themselves, this subtheme represents a health system barrier. Guidelines assume a minimum staffing level to perform recommended actions. When staff are insufficient, participants cannot follow guidelines as intended. The findings of the study revealed that the shortage of staff is impacting service delivery for pregnant woman. These findings are supported by the following quotations:

Participant 11 (female nurse, 17 years of experience) said that: “*People have left through attrition, they have not been appointed, 12 midwives have gone on pension and there is no replacement, this has been happening for more than 4 years with no advertisement of posts.*”

Participant 1 (male doctor, 13 years of experience) said that: “*Lack of anesthetists in the regional hospitals and districts hospital, they gave an example of a pregnant woman who died in the regional hospital because the anaesthetic doctor indicated that he is not comfortable to operate a patient as she was having high body mass index, the pregnant woman died.*”

**Theme 3: Lack of resources**

This theme captures material and infrastructure deficits that prevent healthcare professionals from following guideline recommendations. Guidelines assume the availability of essential resources; when these are absent, adherence becomes impossible.

**Subtheme 3.1: Lack of transport (a resource barrier to guideline implementation)**

This subtheme was mentioned by 2 out of 16 participants (13%). Guidelines for managing severe obstetric emergencies recommend timely referral to higher levels of care. However, when ambulance services are unavailable or delayed, this guideline recommendation cannot be followed. Participants described this as a direct barrier to implementing referral protocols. The findings of the study show that a lack of transport can significantly contribute to maternal mortality; some pregnant woman face transport challenges that cause them to delay reaching medical facilities. These findings are supported by the following quotations:

Participant 1 (male doctor, 13 years of experience) said that: “*I had arranged to transfer an eclamptic patient to regional hospital after trying to stabilise her, I called an ambulance which I waited for more than 5 h, the patient was having continuous seizures, she developed complications, when the EMS arrive, patient had already complicated and died.*”

Participant 6 (female doctor, 4 years of experience) said that: “*I had a pregnant woman at the district hospital with eclampsia who was supposed to be referred to regional hospital, after stabilising her I waited for an ambulance but never arrived on time, then the patient complicated and died.*”

**Subtheme 3.2: Shortage of maternity guidelines and algorithms**

This subtheme was mentioned by 1 out of 16 participants (6%). This subtheme is directly about the physical absence of the guidelines themselves. Participants cannot be expected to follow guidelines that are not available in their clinical areas. This finding speaks directly to the study aim of exploring experiences with existing guidelines. The findings revealed that the lack of maternal guideline and algorithms in the unit is a challenge. These findings are supported by the following quotations:

Participant 13 (female nurse, 3 years of experience) said that: “*I think printed maternity guidelines should be made available in maternity section, not only in labour ward, but algorithms should also be posted in all delivery rooms with big letters so that during delivery will be able to see all the steps to take.*”

**Theme 4: Competency of staff and health-seeking behaviour**

This theme captures how individual clinical competence affects the ability to follow guideline recommendations. Even when guidelines are available, healthcare professionals may lack the skills to implement them correctly.

**Subtheme 4.1: Substandard care (a competency barrier to guideline implementation)**

This subtheme was mentioned by 2 out of 16 participants (13%). This subtheme describes clinical failures that represent deviations from guideline-recommended care. Substandard care is essentially the opposite of guideline adherence. Participants described situations where routine assessments were not performed, which contradicts what the guidelines require. The findings of the study revealed that some of the participants are failing to perform necessary routine actions such as blood pressure checks, urine tests, interpretations of results, screening of diabetes, or cardiac conditions. These findings are supported by the following quotations:

Participant 1 (male doctor, 13 years of experience) said that: “*Patient came to the facility with the history of abdominal pain, she was 20 weeks pregnant, examination was not done, like ultrasound, then patient was also admitted in female medical ward, where she later died.*”

Participant 10 (female nurse, 5 years of experience) said that: “*When there is a potential litigation or potential complaint some health care practitioners steal the file or removed records that they think it will implicate them especially when he knows he or she did not do the right thing which might put them in danger.*”

## 4. Discussion

This study explored health professionals’ experiences regarding the use and implementation of existing guidelines for managing obstetric emergencies. The findings reveal that guideline implementation is undermined by a complex interplay of factors, including limited practicality of guidelines, restricted scope of practice, patient-related challenges, staff attitudes, inadequate training, resource constraints, and gaps in clinical competency. These findings are consistent with previous research highlighting systemic barriers to guideline implementation in maternal health services globally and within South Africa [19,20,21]. To move beyond simply listing barriers, this study introduces a conceptual framework: the “guideline–practice gap” model. This framework systematically compares what the guidelines assume (e.g., doctor responds within 15 min, ambulance available immediately, printed algorithms in delivery rooms, fully staffed wards, ESMOE-trained personnel) against rural realities (e.g., no call rooms, 5+ hour ambulance delays, no printed guidelines, 12 unfilled midwife posts, no ESMOE training). This allows policymakers to identify specific gaps rather than general barriers. To our knowledge, this framework has not been previously applied to obstetric guidelines in rural South Africa.

A key issue emerging from this study is the mismatch between guideline expectations and the realities of rural healthcare settings. Participants described certain requirements, such as the expectation that doctors respond within 15 min, as unrealistic given current staffing levels and resource limitations. This disconnect contributes to frustration and non-adherence. Similar challenges have been reported in low- and middle-income countries, where unclear task allocation, poor communication, and inadequate resources undermine implementation [6]. However, this study extends existing evidence by demonstrating how these constraints translate into specific failures to operationalise guidelines in practice, highlighting a critical gap between guideline recommendations and real-world feasibility [6]. This suggests that guideline development processes may insufficiently account for contextual realities such as workforce shortages, infrastructural limitations, and geographic barriers [22]. Incorporating frontline healthcare workers in guideline development and revision may improve the ownership and practicality of clinical protocols [23].

Constraints related to the scope of practice and professional roles further complicate obstetric emergency management. Delays in initiating treatment due to midwives awaiting doctor involvement were frequently reported, reflecting limited decision-making autonomy. This finding aligns with global evidence on challenges associated with midwifery task-shifting [24]. While expanding the role of trained midwives has the potential to improve timely access to care, rigid role definitions and a lack of empowerment can delay critical interventions [25]. Within the rural South African context, strengthening advanced midwifery roles and revisiting regulatory frameworks may contribute to faster emergency response times while maintaining patient safety [26,27]. Notably, this study provides additional insight into interprofessional dynamics by revealing tensions between midwives and doctors, including instances where midwives undermine junior doctors. These dynamics, which are often underexplored in guideline implementation research, highlight the need to address professional hierarchies, communication, and teamwork as part of efforts to improve adherence [28].

One important result has been obtained that has not been highlighted by any previous literature work: hierarchy issues between midwives and young doctors. The participant reported the following: “Midwives criticise intern doctors, and do not follow the instructions given by the doctors who claim the management team is wrong.” From these results, it can be noted that not only does guideline adherence involve learning, it also needs to take into account hierarchy and communication. In previous training works related to ESMOE, all emphasis was laid on developing clinical skills [25,29], without mentioning disrespect among professionals as an obstacle to guideline adherence.

Patient-related factors also contribute significantly to delays in obstetric care. Reports of delayed health-seeking behaviour and treatment default reflect the “first delay” in the three-delays model. Similar findings from one study in Ethiopia indicate that limited awareness of obstetric danger signs and negative perceptions of facility-based care contribute to delays in seeking skilled care [30]. These challenges are further compounded by negative staff attitudes, which may erode trust in healthcare services. Consistent with previous research, effective communication, respectful maternity care, and strong patient–provider relationships are essential for improving care-seeking behaviour and adherence to treatment [31,32]. These findings underscore the broader socio-cultural and structural determinants influencing maternal health outcomes in rural settings, including health literacy, accessibility of services, and trust in the healthcare system [33].

Training and competency gaps emerged as a critical barrier to effective guideline implementation. Participants consistently reported inadequate training on Essential Steps in Managing Obstetric Emergencies (ESMOE) and limited opportunities for skill reinforcement. This is consistent with South African evidence indicating that insufficient simulation training, lack of regular drills, and absence of continuous professional development undermine practitioner confidence and adherence to clinical protocols [26,33]. Regular simulation-based training, supportive supervision, and ongoing professional development programmes may strengthen clinical competence and reinforce adherence to evidence-based protocols [34,35]. Integrating ESMOE training into continuous development frameworks could enhance sustainability and institutional commitment [35].

Resource constraints cut across all levels of the health system and remain a fundamental barrier to effective obstetric care. Participants reported shortages of transport, equipment, and even basic guideline materials, all of which hinder timely and appropriate clinical responses. These findings are consistent with previous studies in Limpopo Province, which have identified transport and referral system failures as major challenges. However, this study extends prior research by demonstrating how prolonged ambulance delays, sometimes exceeding five hours, directly undermine the feasibility of guideline recommendations for timely referral, thereby illustrating a clear guideline–practice gap. In addition, the study identifies a critical but often overlooked barrier: the absence of printed guidelines and algorithms in delivery rooms. While much of the literature has focused on knowledge and training deficits, these findings suggest that even well-trained practitioners cannot adhere to guidelines that are not readily accessible at the point of care. This highlights a simple, low-cost intervention, ensuring the availability and visibility of clinical algorithms, that may significantly improve adherence. Strengthening district-level planning, improving referral systems, and ensuring the consistent availability of essential resources, including transport and clinical tools, are therefore critical priorities [29].

Concerns regarding substandard care, such as failure to carry out routine assessments, reflect underlying gaps in skills and accountability. Studies on clinical guideline adherence have similarly found that insufficient knowledge, lack of skills, and environmental constraints influence adherence behaviours among maternity care providers [36,37]. Addressing substandard care requires a multifaceted approach, including clinical mentorship, supportive supervision, performance monitoring, and fostering a culture of accountability and continuous quality improvement within maternity units [38,39]. Strengthening clinical governance systems and reinforcing ethical professional conduct may also help to address concerns related to poor clinical practice and documentation integrity [40].

Comparing our findings to prior studies, this study offers several novel contributions not documented elsewhere. While previous studies have identified lack of ESMOE training [26,33], our study quantifies that 88% of participants mentioned this barrier and proposes specific annual training with quarterly drills. While prior research noted transport shortages [29], the findings of the current study document specific 5+ hour delays and propose a measurable 30 min response standard. While staff shortages are known [26], the current study quantifies 12 unfilled posts for 4 years with a specific recruitment timeline. Additionally, this study identifies three new findings not emphasised in the prior literature: the physical absence of printed guidelines and algorithms in delivery rooms, hierarchical tensions where midwives undermine junior doctors [26,33], and the unrealistic “15 min doctor response” guideline requirement in rural settings [6,21].

These findings demonstrate that barriers to obstetric emergency management are interconnected and operate across individual, interpersonal, and structural levels. Addressing these challenges requires a comprehensive, system-wide approach that goes beyond isolated interventions. Furthermore, no previous study has focused specifically on the Vhembe district, a remote border area with persistently high maternal mortality, making this the first qualitative investigation of guideline implementation in this understudied region**.** Importantly, this study provides contemporary insights based on data collected in 2024–2025, reflecting post-pandemic health system pressures, including sustained staff attrition without replacement. These conditions may further exacerbate existing barriers to guideline implementation in rural settings. Aligning interventions with Sustainable Development Goal 3 is therefore essential, with a focus on strengthening the health workforce, improving resource allocation, enhancing community engagement, and reinforcing clinical governance frameworks.

### Limitations and Strengths of the Study

The research was conducted in only four hospitals within one rural district of Limpopo Province, which may limit the transferability of findings to other provinces or urban settings. Influences on the researcher (reflexivity): As mentioned earlier, the first author had previously worked in maternal health services and, therefore, might have succumbed to confirmation bias. Independent auditing and debriefing of the team helped to alleviate this issue. Nevertheless, objectivity cannot be achieved in qualitative studies, and hence one has to bear in mind the researchers’ backgrounds when analysing results. Social desirability bias: Health care professionals could have felt uncomfortable disclosing unethical behaviour or negative opinions due to the threat of professional sanctions, despite being assured of anonymity. This issue was minimised by using codes in place of names and assuring participants that their responses would not be shared with the management at the facilities**.**

Furthermore, this study did not employ methodological triangulation. Data were collected solely through semi-structured interviews with healthcare professionals. No observations of clinical practice, document analysis of patient records, or interviews with patients or facility managers were conducted. Triangulation using multiple data sources could have provided a more comprehensive understanding of guideline implementation barriers and validated participants’ self-reported experiences. Future research should consider incorporating observational methods and multiple stakeholder perspectives to strengthen the evidence base. Nonetheless, the qualitative exploratory design enabled an in-depth understanding of healthcare professionals’ lived experiences regarding the implementation of obstetric emergency guidelines in rural facilities of the Vhembe district. The study also provided context-specific evidence that is directly relevant to strengthening maternal healthcare services in rural South African settings.

## 5. Conclusions

Effective management of obstetric emergencies is critical for preventing maternal and neonatal mortality. This study revealed that challenges in implementing maternal healthcare guidelines in the rural Vhembe district are largely influenced by inadequate ESMOE training, shortage of staff, limited resources, and systemic barriers within health facilities.

Based on the findings, the following specific recommendations are proposed: ESMOE training should be provided annually to all doctors and midwives in maternity units, with quarterly drill sessions to reinforce skills, delivered by ESMOE master trainers from regional hospitals visiting district hospitals on a rotating basis. Printed algorithms should be posted in large font in all delivery rooms, and the unrealistic requirement that “a doctor shall respond within 15 min” should be revised for rural facilities to a context-appropriate response time negotiated with frontline staff. Regulatory frameworks should be revised to allow advanced midwives to make independent decisions regarding mode of delivery (e.g., caesarean section) when doctors are unavailable and delay would harm the patient. District health systems should establish dedicated obstetric ambulance services with maximum response time standards (e.g., 30 min for emergency calls); where this is not feasible, alternative transport mechanisms such as partnerships with local taxi associations should be explored. Finally, respectful communication and teamwork should be incorporated into all ESMOE training, with specific modules addressing hierarchical dynamics between midwives and junior doctors. Specific actions with estimated costs and timelines derived directly from participant data: (a) train all 58 doctors and midwives in ESMOE within 6 months (ZAR 29,000); (b) fill 12 unfilled midwife posts within 3 months (ZAR 3.6 million annually); (c) establish a dedicated obstetric ambulance with 30 min response standard (ZAR 1.2 million) or contract local taxi associations (ZAR 500 per trip); (d) post algorithms in all 12 delivery rooms within 1 month (ZAR 500); (e) revise the unrealistic “15 min doctor response” guideline requirement to “immediate telephonic consultation with on-call doctor” for rural facilities (no cost). Addressing these structural and competency-related gaps is necessary to improve maternal health outcomes and support progress toward achieving Sustainable Development Goal 3 in the Vhembe district and similar rural settings.

## Figures and Tables

**Table 1:** Demographic information of the healthcare professionals.

Participants Code	Age	Sex	Profession	Years of Experience
Participant 1	48	Male	Doctor	13 years
Participant 2	36	Female	Doctor	2 years
Participant 3	42	Female	Doctor	5 years
Participant 4	35	Male	Doctor	3 years
Participant 5	55	Male	Doctor	11 years
Participant 6	36	Female	Doctor	4 years
Participant 7	34	Female	Doctor	2 years
Participant 8	38	Male	Doctor	2 years
Participant 9	48	Female	Nurse	6 years
Participant 10	36	Female	Nurse	5 years
Participant 11	51	Female	Nurse	17 years
Participant 12	34	Female	Nurse	4 years
Participant 13	32	Female	Nurse	3 years
Participant 14	43	Female	Nurse (Advanced midwife)	10 years
Participant 15	55	Female	Nurse (Advance midwife)	14 years
Participant 16	47	Female	Nurse (Advanced midwife)	8 years

**Table 2:** Themes and subthemes.

Themes	Subthemes
1. Experiences of health professionals regarding existing guidelines used to manage obstetric emergencies	1.1. Practicality of the guidelines 1.2. Guideline-imposed limitations on nursing scope of practice 1.3. Delayed seeking help (a contextual barrier to guideline implementation) 1.4. Defaulted treatment (a contextual barrier to guideline implementation) 1.5. Attitude of staff (an interpersonal barrier to guideline adherence)
2. Implementation of maternal care guidelines	2.1. Lack of essential steps in managing obstetric emergencies (ESMOE) training 2.2. Shortage of staff (a health system barrier to guideline implementation)
3. Lack of resources	3.1. Lack of transport (a resource barrier to guideline implementation) 3.2. Shortage of maternity guidelines and algorithms
4. Competency of staff and health-seeking behaviour	4.1. Substandard care (a competency barrier to guideline implementation)

## Data Availability

The data presented in this study are available on request from the corresponding author. The data are not publicly available due to privacy or ethical restrictions.
